# *Gynura procumbens* Root Extract Ameliorates Ischemia-Induced Neuronal Damage in the Hippocampal CA1 Region by Reducing Neuroinflammation

**DOI:** 10.3390/nu13010181

**Published:** 2021-01-08

**Authors:** Woosuk Kim, Hyo Young Jung, Dae Young Yoo, Hyun Jung Kwon, Kyu Ri Hahn, Dae Won Kim, Yeo Sung Yoon, Soo Young Choi, In Koo Hwang

**Affiliations:** 1 Department of Anatomy and Cell Biology, College of Veterinary Medicine, and Research Institute for Veterinary Science, Seoul National University, Seoul 08826, Korea; tank3430@hallym.ac.kr (W.K.); hyoyoung@snu.ac.kr (H.Y.J.); hkinging@snu.ac.kr (K.R.H.); ysyoon@snu.ac.kr (Y.S.Y.); 2 Department of Biomedical Science and Research Institute for Bioscience and Biotechnology, Hallym University, Chuncheon 24252, Korea; 3 Department of Anatomy, College of Medicine, Soonchunhyang University, Cheonan 31151, Korea; dyyoo@sch.ac.kr; 4 Department of Biochemistry and Molecular Biology, Research Institute of Oral Sciences, College of Dentistry, Gangneung-Wonju National University, Gangneung 25457, Korea; donuts25@gwnu.ac.kr (H.J.K.); kimdw@gwnu.ac.kr (D.W.K.)

**Keywords:** *Gynura procumbens* root extract, neuroprotection, pro-inflammatory cytokines, microglia, ischemia

## Abstract

*Gynura procumbens* has been used in Southeast Asia for the treatment of hypertension, hyperglycemia, and skin problems induced by ultraviolet irradiation. Although considerable studies have reported the biological properties of *Gynura procumbens* root extract (GPE-R), there are no studies on the effects of GPE-R in brain damages, for example following brain ischemia. In the present study, we screened the neuroprotective effects of GPE-R against ischemic damage and neuroinflammation in the hippocampus based on behavioral, morphological, and biological approaches. Gerbils received oral administration of GPE-R (30 and 300 mg/kg) every day for three weeks and 2 h after the last administration, ischemic surgery was done by occlusion of both common carotid arteries for 5 min. Administration of 300 mg/kg GPE-R significantly reduced ischemia-induced locomotor hyperactivity 1 day after ischemia. Significantly more NeuN-positive neurons were observed in the hippocampal CA1 regions of 300 mg/kg GPE-R-treated animals compared to those in the vehicle-treated group 4 days after ischemia. Administration of GPE-R significantly reduced levels of pro-inflammatory cytokines such as interleukin-1β, -6, and tumor necrosis factor-α 6 h after ischemia/reperfusion. In addition, activated microglia were significantly decreased in the 300 mg/kg GPE-R-treated group four days after ischemia/reperfusion compared to the vehicle-treated group. These results suggest that GPE-R may be one of the possible agents to protect neurons from ischemic damage by reducing inflammatory responses.

## 1. Introduction

Transient interruption of blood supply to the brain, referred to as transient forebrain ischemia, causes neuronal loss in the striatum, somatosensory cortex, and hippocampus [1,2]. Ischemic damage manifests as different phenotypes depending on the time post-injury and the affected regions. Mongolian gerbil (*Meriones unguiculatus*) is one of the most useful animals to induce transient forebrain ischemia because of its incomplete connections between basilar and internal carotid arteries at the base of the brain [3,4]. In this animal, damage to the hippocampus causes locomotor hyperactivity one day after ischemia/reperfusion and cognitive impairments by neuronal death in the pyramidal cells four days after ischemia [5,6,7,8,9]. The transient ischemia and subsequent reperfusion significantly increase the oxygen free radicals and induce neuronal damage in the hippocampus [10,11]. In addition, neuroinflammation induced by ischemic neuronal death activates microglia in the affected regions, which secrete pro-inflammatory cytokines such as interleukin (IL)-1β, IL-6, and tumor necrosis factor-α (TNF-α) [12,13]. Increased expression of IL-1β and TNF-α can facilitate the disruption of the blood-brain barrier and accelerate the formation of edema in the brain [14]. Many individuals suffer from transient forebrain ischemia each year, and survivors are often left with disabilities including long-term sensorimotor deficits and cognitive impairment [15,16]. However, to date, there is no effective treatment for transient forebrain ischemia [17].

*Gynura procumbens* belongs to the family of Asteraceae and its leaves have been used in folk medicine for the improvement of various types of illnesses and diseases [18] because it is non-toxic when consumed [19]. For example, an extract from *Gynura procumbens* leaves (GPE-L) decreases blood glucose levels and systolic blood pressure [20,21,22,23,24] and significantly reduces heart rate and atrial contractility [25,26]. In addition, GPE-L has antioxidant effects that reduce the generation of ultraviolet irradiation-induced reactive oxygen species and the expression of metalloproteinases in human dermal fibroblasts [27]. Whole plant extract of *Gynura procumbens* reduces the oxidative stress and damage in membrane by modulating the antioxidants [28]. However, in a recent study, GPE-L showed no reduction of oxidative stress and very low antioxidant activity in in vitro assay [18]. Another recent study showed that GPE petroleum ether extract could reduce the formation of oxygen radical species in vessels by inducing the vasodilation [29]. An extract from *Gynura procumbens* roots (GPE-R) with abundant content of phenolic, flavonoid compounds, and ascorbic acid, has shown higher antioxidant capacities than GPE-L [18], suggesting that it may also have therapeutic benefits. In addition, treatment with GPE-L facilitates the healing process in tissue wounds by reducing inflammatory processes [30]. Despite GPE having been studied for its biological effects, including antioxidant and anti-inflammatory effects, there have been no studies on GPE effects on cellular damage induced by transient forebrain ischemia [31].

In this study, we examined the potential ameliorative effects of GPE-R on neuronal damage in Mongolian gerbils, at 4 days after inducing transient forebrain ischemia.

## 2. Materials and Methods

### 2.1. Experimental Animals

Sixty male gerbils (6 ± 0.5 months of age; 67 ± 4.3 g body weight) were used in the study. Animals were purchased from Japan SLC Inc. (Shizuoka, Japan) and mated each other. Only male gerbils were selected because they show larger infarct size, higher inflammatory responses, and increased activation of microglia after the ischemic insult [32]. Animals were housed in cages (four gerbils per cage) in a conventional area under standard conditions as described previously [33,34]. The experimental procedure was conducted according to international standards [35] and the protocol was approved by ethical committee (SNU-170807-11).

### 2.2. Preparation of GPE-R

For GPE-R, roots of *Gynura procumbens* (100 g) were purchased from a local oriental herb market (Kyung Dong Si Jang, Seoul, Korea). Roots of *Gynura procumbens* were ground, dried, and macerated using 80% ethanol (3 times) for 72 h at 20 °C, and washed with distilled water at 20 °C for 2 h. The extract was filtered using Whatman No.1 filter paper (Whatman, Maidstone, UK) and the insoluble materials were eliminated by centrifugation at 10,000× *g* for 30 min at 20 °C. The residual solvent was removed using rotary evaporator at 40 °C and the extract was freeze-dried. The final yield of was 9.49%.

### 2.3. Administration of GPE-R

The gerbils were divided into four groups: control group, vehicle-treated ischemic group, 30 mg/kg GPE-R-treated ischemic group, and 300 mg/kg GPE-R-treated ischemic group. Gerbils received oral administration of distilled water (vehicle-treated ischemic group) or GPE-R (30 mg/kg and 300 mg/kg GPE-R-treated ischemic groups) with a feeding needle once a day for 3 weeks before the surgery. Doses of GPE-R were chosen based on previous findings which showed that administration of 150–450 mg/kg of GP aqueous extract improved fertility [36] and administration of 25–50 mg/kg of GP stem extract (GPE-S) ameliorated ethanol-induced steatosis [37].

### 2.4. Ischemic Surgery

Ischemic surgery was conducted as described previously [32,33]. The animals were anesthetized with a mixture of 2.5% isoflurane (Hana Pharmaceutical Co., Ltd., Seoul, Korea) [38,39] in 33% oxygen and 67% nitrous oxide 1 h after the last vehicle or GPE-R treatment. The ventral neck region was shaved and a midline incision was made. Common carotid arteries were freed from the vagosympathetic trunk and isolated with Chromic Catgut #4. Occlusion of both arteries was done by non-traumatic aneurysm clips for 5 min and reperfusion was confirmed to observe the current flow of retinal central artery using an ophthalmoscope (HEINE K180^®^; Heine Optotechnik, Herrsching, Germany). Bilateral common carotid occlusion was not performed in the control group (sham-operated). Animals after ischemic surgery were kept on the thermal incubator to maintain the body temperature without treatment with analgesics until they were euthanized because opioid agonists, anti-inflammatory drugs, and dexmedetomidine show neuroprotection against ischemic damage [40,41,42,43]. In addition, acetaminophen protects the brain tissue after middle cerebral artery occlusion, and thus should not be used to induce analgesia in rodents subjected to experimental stroke [43]. After ischemic surgery, animals were carefully monitored at least 4 times a day to identify animal welfare before reaching humane endpoint as described by Percie du Sert et al. [44]. Animals were sacrificed when the animals showed reduction of body weight (>20%), complete anorexia for 24 h, partial anorexia (<50% food intake) for 3 days, and inability and severe reluctance to stand for 24 h. In addition, animals were sacrificed when the animals show severe depression, lack of movement, and hypothermia.

### 2.5. Locomotor Activity

To observe the effects of GPE-R on locomotor hyperactivity caused by striatum damage, locomotor activity was traced for 60 min before and 1 day after ischemia, as described previously [33]. The apparatus was made of Plexiglas (25 × 20 × 12 cm) and locomotor activity was recorded with Photobeam Activity System-Home Cage (San Diego Instruments, San Diego, CA, USA).

### 2.6. Tissue Processing

Following spontaneous motor activity observation, animals were deeply anesthetized with a mixture of alfaxalone (Alfaxan, 75 mg/kg; Careside, Seongnam, Korea) and xylazine (10 mg/kg; Bayer Korea, Seoul, Korea) 4 days after ischemia/reperfusion as a modified method [38,39,45] because neuronal cell death and subsequent glial activation was mainly detected in the hippocampal CA1 region 4 days after ischemia. Thereafter, brain samples were obtained by transcardial perfusion with 0.1 M phosphate-buffered saline (pH 7.4), followed by 4% paraformaldehyde in 0.1 M phosphate-buffer (pH 7.4), as described previously [33,34]. Brain tissue was cryoprotected by overnight saturation with 30% sucrose. Coronal brain slices (30 μm) located between 1.4 and 2.0 mm caudal to bregma in reference to a stereotaxic brain gerbil atlas [46], were obtained using a cryostat (Leica, Wetzlar, Germany). Five slices at 90-μm intervals were immunohistochemically processed with the conventional avidin-biotin complex method and visualized with 3,3′-diaminobenzidine tetrachloride (Sigma). Primary antibodies were used as follows: mouse anti-neuronal nuclei (NeuN) antibody (1:1000; EMD Millipore, Temecula, CA, USA) and rabbit anti-ionized calcium-binding adapter molecule 1 (Iba-1; 1:500; Wako, Osaka, Japan).

The number of NeuN-positive nuclei in the dorsal hippocampus was measured in the hippocampal CA1 region using an image analysis system (software: Optimas 6.5, CyberMetrics, Scottsdale, AZ, USA). Analysis of the Iba-1 immunoreactivity in the hippocampal CA1 region was assessed by the optical density in 256-pixel gray levels. The hippocampal CA1 region was divided into 3 subregions such as stratum pyramidale, oriens, and radiatum. The optical density was measured in each subregion using ImageJ v. 1.80 software (National Institutes of Health) and results were transformed to a relative optical density (ROD) = log_10_(256/mean gray level) as described in previous studies [33,34].

### 2.7. ELISA for Pro-Inflammatory Cytokines

To measure changes in TNF-α, IL-1β, and IL-6 levels in the hippocampus, separate animals from the control, vehicle-treated ischemic, 30 mg/kg GPE-R-treated ischemic, and 300 mg/kg GPE-R-treated ischemic groups (*n* = 7 per group) were sacrificed with deep anesthesia with alfaxalone (100 mg/kg) and xylazine (15 mg/kg) and subsequent decapitation, and the extracted brains were used for enzyme-linked immunosorbent assay (ELISA) as described previously by our colleagues [47] with commercial kits (EMD Millipore, Billerica, MA, USA).

### 2.8. Statistical Analysis

Data were analyzed to check for significant differences between groups by one-way analyses of variance, followed by Bonferroni’s post-hoc tests using GraphPad Prism 5.01 software (GraphPad Software, Inc., La Jolla, CA, USA). Level of significance was set at *p* < 0.05.

## 3. Results

### 3.1. Body Weights

**Figure 1:**
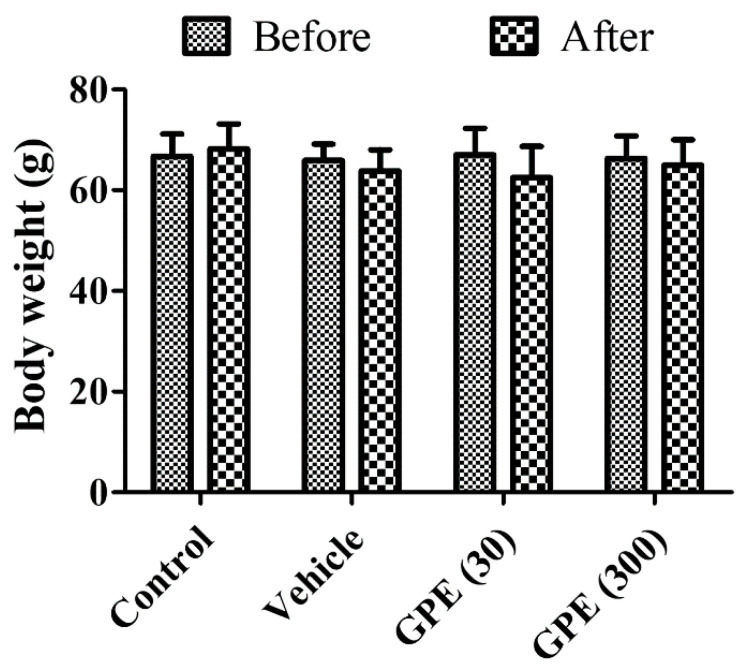
Body weight in the control, vehicle-treated ischemic (Vehicle), 30 mg/kg *Gynura procumbens* roots (GPE-R)-treated ischemic (GPE30), and 300 mg/kg GPE-R-treated ischemic (GPE300) groups. There are no significant changes in body weight before and after ischemia or GPE-R treatment. Error bars represent standard errors of the means.

Gerbils showed similar body weight (65.93–67.04 g) before ischemia and body weight was slightly increased (68.19 g) in the control group, but ischemia-operated gerbils showed slightly decreases in body weight (62.48–64.95 g). However, there were no significant differences in body weights before and 4 days after ischemia in all groups (Figure 1).

### 3.2. Effects of GPE-R on Locomotor Activity before and 1 Day after Ischemia

**Figure 2:**
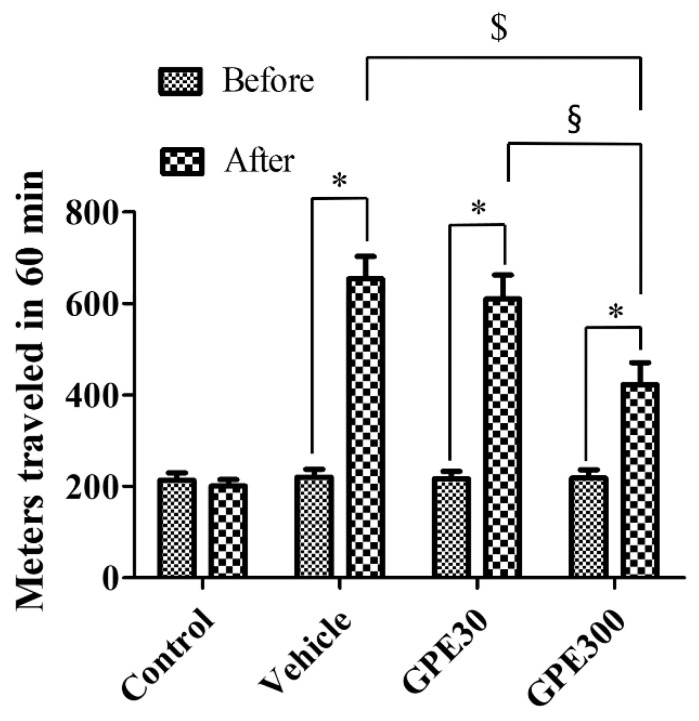
Locomotor activity in the control, vehicle-treated ischemic (Vehicle), 30 mg/kg GPE-R-treated ischemic (GPE30), and 300 mg/kg GPE-R-treated ischemic (GPE300) groups. The total distance (meters) traveled in 60 min was observed 1 day before and after ischemia/reperfusion (*n* = 7 per group, * *p* < 0.05 vs. the control group; ^$^ *p* < 0.05 vs. the vehicle group; ^§^ *p* < 0.05 vs. the GPE30 group). Error bars represent standard errors of the means.

The distance traveled was measured to compare the locomotor hyperactivity among the groups, both before and one day after ischemia/reperfusion. In the control group, spontaneous motor activity showed no significant difference before and after sham surgery. In the vehicle-treated ischemic and 30 mg/kg GPE-R-treated ischemic groups, gerbils showed significantly higher locomotor hyperactivity 1 day after ischemia compared to one day before. In the vehicle-treated ischemic and 30 mg/kg GPE-R-treated ischemic groups, the distance traveled was 2.97-fold and 2.82-fold one day after vs. before ischemia, respectively. In the 300 mg/kg GPE-R-treated ischemic group, locomotor activity was increased one day after ischemia; however, it showed significant lower levels compared to that in the vehicle-treated ischemic group (1.94-fold one day after ischemia vs. one day before ischemia, *p* < 0.001) (Figure 2).

### 3.3. Effects of GPE-R on Ischemia-Induced Neuronal Death 4 Days after Ischemia

**Figure 3:**
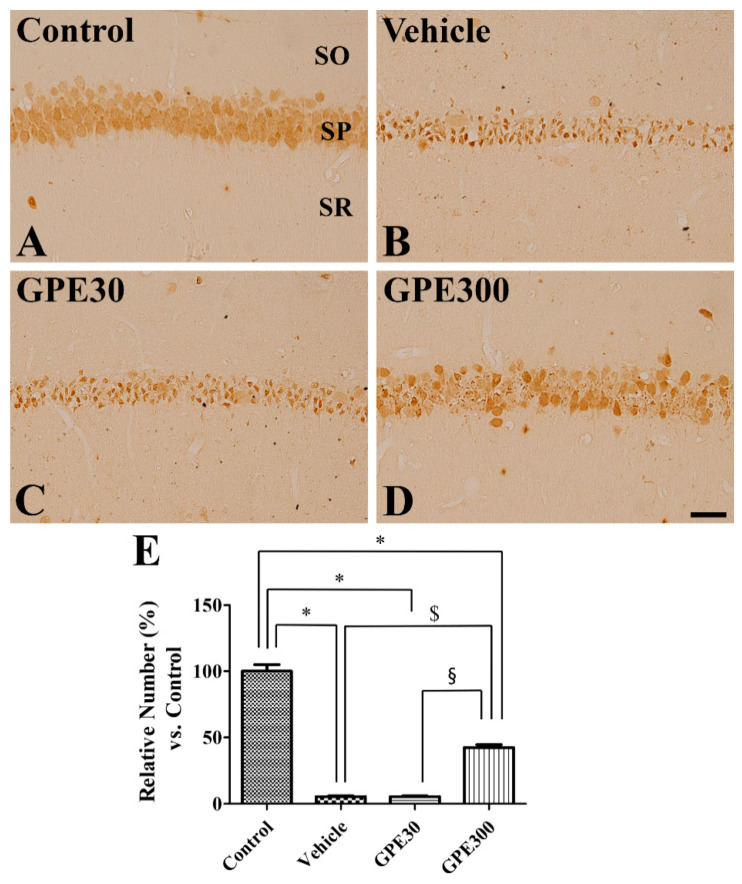
Immunohistochemistry for NeuN in the CA1 region of the control (**A**), vehicle-treated ischemic (Vehicle, **B**), 30 mg/kg GPE-R-treated ischemic (GPE30, **C**), and 300 mg/kg GPE-R-treated ischemic (GPE300, **D**) groups. SO, stratum oriens; SP, stratum pyramidale; SR; stratum radiatum. Scale bar = 50 μm. (**E**) The number of NeuN-immunoreactive neurons per section in the hippocampal CA1 region of each group compared with the control group, is shown (*n* = 7 per group, * *p* < 0.05 vs. the control group; ^$^ *p* < 0.05 vs. the vehicle group; ^§^ *p* < 0.05 vs. the GPE30 group). Error bars represent standard errors of the means.

In the control group, many NeuN-positive neurons were detected in the CA1 region four days after sham surgery (Figure 3A). In the vehicle-treated ischemic and 30 mg/kg GPE-R-treated ischemic groups, only a few NeuN-positive neurons were observed in the CA1 region four days after ischemia/reperfusion and most surviving neurons were identified as non-pyramidal cells, based on morphology and location (Figure 3B,C). In these groups, the percentage of NeuN-immunoreactive neurons was 5.56% and 5.50% of the control group, respectively (Figure 3E). In the 300 mg/kg GPE-R-treated ischemic group, some NeuN-positive neurons were observed in the stratum pyramidale of CA1 region (Figure 3D) and the number of NeuN-immunoreactive neurons was significantly increased to 42.47% of the control group compared to that in the vehicle-treated group (*p* < 0.001) (Figure 3E).

### 3.4. Effects of GPE-R on Ischemia-Induced Activation of Microglia 4 Days after Ischemia

**Figure 4:**
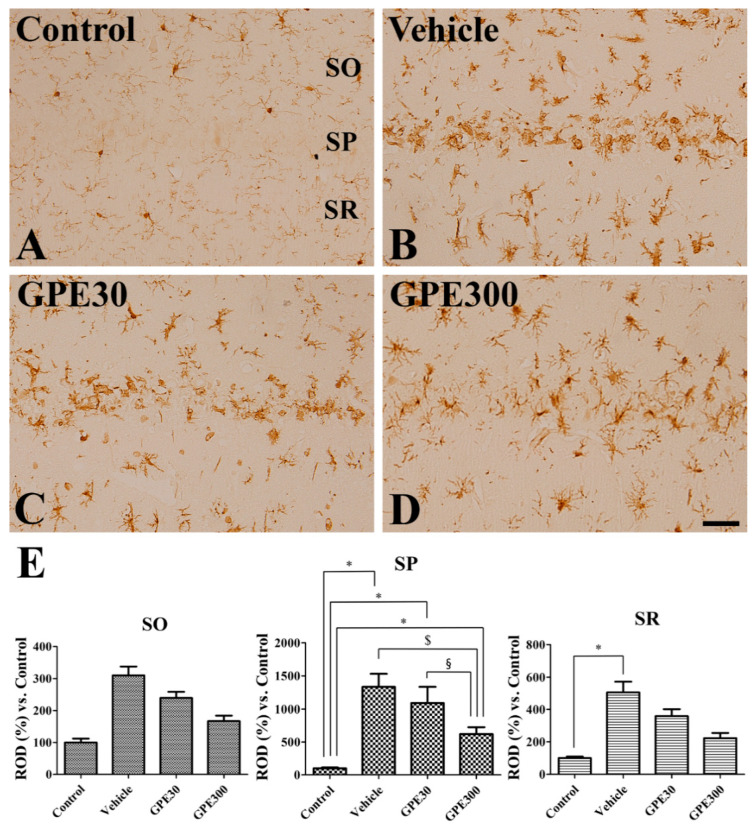
Immunohistochemistry for Iba-1 in the CA1 region of the control (**A**), vehicle-treated ischemic (Vehicle, **B**), 30 mg/kg GPE-R-treated ischemic (GPE30, **C**), and 300 mg/kg GPE-R-treated ischemic (GPE300, **D**) groups. SO, stratum oriens; SP, stratum pyramidale; SR; stratum radiatum. Scale bar = 50 μm. (**E**) ROD per section in the SO, SP, and SR of hippocampal CA1 region in each group compared with the control group, is shown (*n* = 7 per group, * *p* < 0.05 vs. the control group; ^$^ *p* < 0.05 vs. the vehicle group; ^§^ *p* < 0.05 vs. the GPE30 group). Error bars represent standard errors of the means.

In the control group, Iba-1 immunoreactive microglia were diffusely found in the CA1 region and had a morphology of a round soma with thin processes (Figure 4A). In the vehicle-treated ischemic and 30 mg/kg GPE-R-treated ischemic groups, Iba-1 immunoreactivity was showed in microglia, which had hypertrophied somas with very short or no processes (phagocytic form) in the stratum pyramidale, while in the stratum oriens and radiatum, they showed hypertrophied somas with bushy processes (reactive form) (Figure 4B,C). In these groups, Iba-1 immunoreactivity was significantly increased in the stratum pyramidale of CA1 region to 1334.1 and 1094.5% of the control group, respectively (Figure 4E). In the stratum radiatum of CA1 region, Iba-1 immunoreactivity was significantly increased only in the vehicle-treated group compared to that in the control group, while in the stratum oriens, there were no significant differences on Iba-1 immunoreactivity among groups (Figure 4E). In the 300 mg/kg GPE-R-treated ischemic group, there were few Iba-1-immunoreactive microglia in the phagocytic form in the stratum pyramidale, although many reactive microglia were present in the stratum oriens and radiatum (Figure 4D). In this group, significantly lower levels of Iba-1 immunoreactivity were detected in the stratum pyramidale of CA1 region compared to the vehicle-treated ischemic group (621.48% of the control group) (Figure 4E).

### 3.5. Effects of GPE-R on ISCHEMIA-Induced Release of Pro-Inflammatory Cytokines 6 h after Ischemia

**Figure 5:**
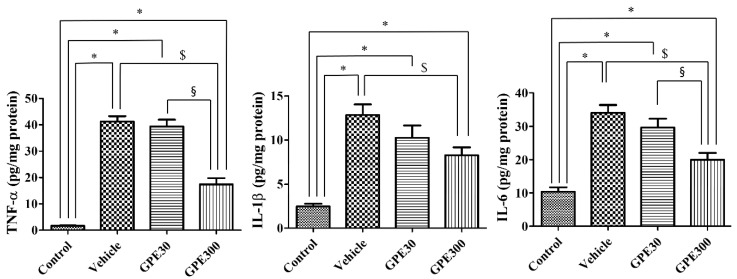
Levels of tumor necrosis factor-α (TNF-α), interleukin (IL)-1β, and IL-6 in the hippocampi of the control, vehicle-treated ischemic (Vehicle), 30 mg/kg GPE-R-treated ischemic (GPE30), and 300 mg/kg GPE-R-treated ischemic (GPE300) groups (*n* = 7 per group, * *p* < 0.05 vs. the control group; ^$^ *p* < 0.05 vs. the vehicle group; ^§^ *p* < 0.05 vs. the GPE30 group) are shown. Error bars represent standard errors of the means.

Six hours after ischemia/reperfusion, IL-6, IL-1β, and TNF-α levels in the vehicle-treated ischemic group were significantly increased in hippocampal homogenates compared to the control group. IL-6, IL-1β, and TNF-α levels in the vehicle-treated ischemic group were 3.30-, 5.17-, and 24.72-fold of those in the control group, respectively. In the 30 mg/kg GPE-R-treated ischemic group, IL-6 (*p* = 0.2477), IL-1β (*p* = 0.2009), and TNF-α (*p* = 0.5792) levels in the hippocampus were similar to those of the vehicle-treated ischemic group. In the 300 mg/kg GPE-R-treated ischemic group, IL-6 (*p* = 0.0019), IL-1β (*p* = 0.0165), and TNF-α (*p* < 0.0001) levels were significantly decreased to about half of those of the vehicle-treated group. IL-6 (*p* = 0.0206) and TNF-α (*p* = 0.0003) levels were significantly decreased in 300 mg/kg GPE-R-treated ischemic group compared to that in the 30 mg/kg GPE-R-treated ischemic group, while IL-1β (*p* = 0.2706) levels did not show any significant changes between 30 and 300 mg/kg GPE-R-treated ischemic group (Figure 5).

## 4. Discussion

Brain ischemia is the most severe type of stroke, and many studies have investigated its pathophysiology, molecular mechanisms, and potential therapeutic agents [48,49]. However, most studies have failed due to toxicity and side effects [17]. Medicinal plants have been studied as alternative preventive or therapeutic agents in various diseases including brain ischemia because of their minimal toxic effects [31,50,51,52].

In the present study, we selected GPE-R as a possible candidate to prevent the neuronal damage induced by ischemia, because GPE has various functional components including chlorogenic acid, quercetin-3-0-rhamnosyl, kaempferol-3-0-glucoside, and kaempferol-3-0-rutinoside [37,53]. We assessed the neuroprotective effects of GPE-R in gerbils one day after ischemia based on their total distance traveled, which had been proved to be increased after ischemic hippocampal cell damage in previous studies [54,55]. However, the locomotor activity is decreased to basal levels seven days after ischemia [56,57]. Hence, the locomotor activity test is a useful predictable model to assess hippocampal damage early [57]. Administration of 300 mg/kg GPE-R significantly reduced the distance traveled one day after ischemia/reperfusion compared to both vehicle-treated ischemic and 30 mg/kg GPE-R-treated ischemic groups. We also confirmed the neuroprotective effects of GPE-R against ischemic damage in the hippocampal CA1 region by NeuN immunohistochemistry. Most pyramidal cells showed neuronal death and surviving neurons were relatively few (about 4–10% in total). In this regard, we conducted NeuN staining to detect surviving neurons in the hippocampal CA1 region. Administration of 300 mg/kg, but not 30 mg/kg, GPE-R significantly reduced CA1 neuronal death four days after ischemia. GPE has high phenolic content and is a potential source of treatment for various diseases [19]. Furthermore, extracts from GPE-S mitigated ethanol-induced lipid accumulation in the livers of mice [37] and treatment with GPE-L significantly ameliorated ultraviolet B-induced matrix metalloproteinase-1 (MMP-1) expression in the culture medium of human dermal fibroblasts [27]. However, this study provides new insight that GPE-R has in vivo neuroprotective effects against ischemic damage in gerbils, although one poster presentation demonstrated the neuroprotective effects of GPE against glutamate-induced neurotoxicity in HT22 cells by modulation of intracellular calcium, reactive oxygen species levels, and mitochondrial membrane potential level [58].

Ischemia-induced reactive microglia produce and release neurotoxic molecules and pro-inflammatory cytokines [59,60]. In the present study, administration of 300 mg/kg GPE-R significantly reduced the phagocytic form of microglia in the stratum pyramidale of CA1 as well as the reactive form of microglia in the stratum oriens and radiatum. In addition, we observed the significant reduction of pro-inflammatory cytokines such as IL-6, IL-1β, and TNF-α in hippocampal homogenates 6 h after ischemia. We observed the levels of IL-6, IL-1β, and TNF-α in the hippocampus 6 h after ischemia because these pro-inflammatory cytokines are increased biphasically, with a prominent increase at this early period after ischemia [61,62]. In addition, nuclear factor κB (NF-κB) is a transcription factor involved in the regulation of the induction of pro-inflammatory genes such as IL-1, IL-2, IL-6, IL-8, and TNF-α, and functions in both innate and adaptive immune cells [63]. GPE has been shown to inhibit the nuclear transfer of NF-κB [64] as well as the release of pro-inflammatory cytokines such as IL-6 and IL-8 in human HaCat keratinocytes [27]. GPE also decreases inflammatory response stimulated by lipopolysaccharide (LPS) by inhibiting nitric oxide production and inducible nitric oxide synthase expression in RAW 264.7 cells [65]. Essential oils from *Gynura procumbens* have shown anti-inflammatory effects by inhibiting overexpression of COX-2 and reducing migration of RAW 264.7 macrophages in cell culture [66]. In addition, GPE also has vasodilatory effects via the inhibition of angiotensin converting enzyme [22], bradykinin [67], and calcium channel [68], which play an important role in neuronal death induced by ischemia in gerbils [69,70].

Another possible mechanism implicated in GPR neuroprotective activity against ischemic damage is functional component-mediated neuroprotection. Indeed, GPE contains chlorogenic acid, quercetin-3-0-rhamnosyl, kaempferol-3-0-glucoside, and kaempferol-3-0-rutinoside [37,53]. Intraperitoneal injection of 30 mg/kg chlorogenic acid reduces brain and blood-brain barrier damage by inhibition of MMP-2 and MMP-9 [71]. Kaempferol protects neurons from ischemic damage by reducing nitrosative-oxidative stress, caspase-9 activity, and poly-(ADP-ribose) polymerase degradation [72]. In addition, quercetin ameliorates neuronal death induced by focal and transient forebrain ischemia via anti-oxidative and anti-inflammatory pathways [73,74]. However, the active ingredients responsible for the neuroprotective effects of GPE-R are still known and remain to be elucidated.

In summary, the pretreatment with GPE-R significantly promotes the survival of neurons in the hippocampal CA1 region after ischemia/reperfusion by reducing reactive microglia and pro-inflammatory cytokines such as IL-6, IL-1β, and TNF-α. GPE-R can be a candidate functional food to protect neurons from ischemic damage.

## Data Availability

The datasets and supporting materials generated during and/or analyzed during the current study are available from the corresponding author on reasonable request.
